# NADH and NADPH peroxidases as antioxidant defense mechanisms in intestinal sulfate-reducing bacteria

**DOI:** 10.1038/s41598-023-41185-3

**Published:** 2023-08-25

**Authors:** Ivan Kushkevych, Dani Dordević, Mohammad I. Alberfkani, Márió Gajdács, Eszter Ostorházi, Monika Vítězová, Simon K.-M. R. Rittmann

**Affiliations:** 1 Department of Experimental Biology, Faculty of Science, Masaryk University, Kamenice 753/5, 62500 Brno, Czech Republic; 2 Department of Plant Origin Food Sciences, Faculty of Veterinary Hygiene and Ecology, University of Veterinary Sciences Brno, Palackého tř. 1946/1, 612 42 Brno, Czech Republic; 3 Department of Medical Laboratory Technology, College of Health and Medical Techniques, Duhok Polytechnic University, Duhok, Kurdistan Region Iraq; 4 Department of Oral Biology and Experimental Dental Research, Faculty of Dentistry, University of Szeged, Tisza Lajos Krt. 64-66., 6720 Szeged, Hungary; 5 Faculty of Medicine, Institute of Medical Microbiology, Semmelweis University, Nagyvárad Tér 4, 1089 Budapest, Hungary; 6 Archaea Physiology & Biotechnology Group, Department of Functional and Evolutionary Ecology, Universität Wien, 1090 Wien, Austria

**Keywords:** Microbiology, Biochemistry, Enzymes

## Abstract

Animal and human feces typically include intestinal sulfate-reducing bacteria (SRB). Hydrogen sulfide and acetate are the end products of their dissimilatory sulfate reduction and may create a synergistic effect. Here, we report NADH and NADPH peroxidase activities from intestinal SRB *Desulfomicrobium orale* and *Desulfovibrio piger*. We sought to compare enzymatic activities under the influence of various temperature and pH regimes, as well as to carry out kinetic analyses of enzymatic reaction rates, maximum amounts of the reaction product, reaction times, maximum rates of the enzyme reactions, and Michaelis constants in cell-free extracts of intestinal SRB, *D. piger* Vib-7, and *D. orale* Rod-9, collected from exponential and stationary growth phases. The optimal temperature (35 °C) and pH (7.0) for both enzyme’s activity were determined. The difference in trends of Michaelis constants (*K*_m_) during exponential and stationary phases are noticeable between *D. piger* Vib-7 and *D. orale* Rod-9; *D. orale* Rod-9 showed much higher *K*_m_ (the exception is NADH peroxidase of *D. piger* Vib-7: 1.42 ± 0.11 mM) during the both monitored phases. Studies of the NADH and NADPH peroxidases—as putative antioxidant defense systems of intestinal SRB and detailed data on the kinetic properties of this enzyme, as expressed by the decomposition of hydrogen peroxide—could be important for clarifying evolutionary mechanisms of antioxidant defense systems, their etiological role in the process of dissimilatory sulfate reduction, and their possible role in the development of bowel diseases.

## Introduction

Sulfate-reducing bacteria (SRB) develop rapidly in the presence of lactate and sulfate in the human gut, which leads to the buildup of hydrogen sulfide (H_2_S), which is toxic and damaging to epithelial intestinal cells^1–8^. However, H_2_S is a sulfur source for methanogenic archaea that are also detected in high abundance in the gastrointestinal tract. Moreover, it might be possible that the overproduction and accumulation of H_2_S may be damaging and not its mere presence^9, 10^. Inflammatory bowel illnesses (IBDs) may develop in humans and animals due to an increase in the number of SRB and the intensity of dissimilatory sulfate reduction in the gut^2, 11–16^.

Inorganic sulfate or other oxidized forms of sulfur are converted to sulfide by dissimilatory SRB^17–19^. Since these bacterial communities are heterotrophs, they need a supply of organic carbon. Simple organic compounds like lactate, pyruvate, and malate can serve as this carbon source for *Desulfovibrio* and *Desulfomicrobium* species^18, 20^; these are subsequently oxidized to acetate with the concurrent reduction of sulfate to sulfide^1, 21, 22^. Through this multistage process of oxidation for organic molecules heterotrophs obtain their cellular energy^23–26^. SRB species typically employ lactate as a substrate, which they then oxidize into acetate using pyruvate, simultaneously^24^.

Endogenous antioxidant enzymes may act in removing reactive oxygen species (ROS). The well-known antioxidant enzymes that prevent intracellular ROS production and lipid peroxidation include superoxide dismutase (SOD), catalase, and peroxidases^27^. While catalase breaks down hydrogen peroxide (H_2_O_2_) via dismutation into water and molecular oxygen (O_2_), SOD transforms superoxide radicals into hydrogen peroxide (H_2_O_2_)^28^.

Peroxidases (EC 1.11.1.7) are heme-containing enzymes that use H_2_O_2_ to catalyze the oxidation of a range of substrates^29^.

A non-radical ROS byproduct of typical aerobic metabolism is H_2_O_2_. The high levels of H_2_O_2_ could, however, be transformed into other, more reactive ROS, like hydroxyl radicals, which can oxidize biomolecules and cause aging, cell death, tissue damage, cardiovascular disorders, and malignant transformation. Therefore, a crucial component of all antioxidant activity in a biological system is H_2_O_2_ scavenging activity^30^. H_2_O_2_ and other hydrogen acceptors are where peroxidase is more active in comparison with other electron acceptors. Ascorbate, a number of amino acids, and polyphenolic substances are hydrogen donors to SRB^31^:

$${\text{H}}_{{2}} {\text{O}}_{{2}} + {\text{Hydrogen}}\;{\text{Donor}}\;\mathop{\longrightarrow}\limits^{{{\text{Peroxidases}}}}\;{\text{H}}_{{2}} {\text{O}} + {\text{Oxidized}}\;{\text{Donor}}$$

NADH peroxidase (EC 1.11.1.1) is responsible for the enzymatic catalysis of the following chemical reaction^32–35^:

$${\text{NADH }} + {\text{ H}}^{ + } + {\text{ H}}_{{2}} {\text{O}}_{{2}} \rightleftharpoons {\text{NAD}}^{ + } + {\text{ 2 H}}_{{2}} {\text{O}}$$

The function of NADH peroxidase is to inactivate H_2_O_2_ that is generated within the cell, by glycerol-3-phosphate oxidase during the metabolism of glycerol and the dismutation of superoxide, before the H_2_O_2_ could cause damage to essential cellular components. NADH eliminates potentially toxic H_2_O_2_ under aerobic growth conditions and this process represents an enzymatic defense against H_2_O_2_-mediated oxidative stress. The enzyme may also protect against exogenous H_2_O_2_ and in that way contribute to bacterial virulence^32–34,36^. A NADPH peroxidase (EC 1.11.1.2) is an enzyme that catalyzes H_2_O_2_ by the same chemical reaction like NADH peroxidase, but instead of NAD^+^ is NADP^+^ formed^37^.

NADH and NADPH peroxidases have been described in sulfate-reducing *Desulfovibrio* bacteria lacking catalase activity (except *D. desulfuricans* ATCC 27774)^38^. Active NADH and NADPH peroxidases catalyze the cleavage of H_2_O_2_ in the *D. desulfuricans* cells. These enzymes were found in both the periplasm and cytoplasm membrane-containing fractions. NADPH peroxidase is mainly localized in the periplasm (70%), whereas NADH peroxidase is nearly equally distributed in the periplasm and in the cytoplasm membrane-containing fractions (60 and 40%, respectively)^38^. The activity of peroxidase does not depend on the physiological state of the culture. On the other hand, NADH peroxidase activity of the cells in the stationary growth phase is higher than the enzyme activity in cells during the exponential growth phase^38^. It should be emphasized that peroxidases from intestinal SRB *D. piger* and *D. orale* have never been well-characterized. There are some data on peroxidases in different organisms as well as in SRB isolated from the environment in the literature that is currently available^27,28,30,38^. However, information on the activity and kinetics of this enzyme from intestinal SRB *D. piger* and *D. orale* has not yet been reported.

The current study's objectives were to determine the peroxidase activity in cell-free extracts of the intestinal sulfate-reducing bacteria *D. piger* Vib-7 and *D. orale* Rod-9 as a major antioxidant system enzyme and to conduct kinetic assessments of their enzymatic activities.

## Materials and methods

### Bacterial culture and cell-free extracts preparation

The sulfate-reducing bacteria *Desulfovibrio piger* Vib-7 (GenBank: KT881309.1) and *Desulfomicrobium orale* Rod-9 (GenBank: MF939896), which were isolated from feces of a healthy human's large intestine and identified using the sequence analysis of the 16S rRNA gene, were the focus of this study^20, 39^.

In a modified liquid Postgate's anoxic medium C, both bacteria were grown^20, 39–41^. In order to create an O_2_-free medium, the medium was boiled in water for 30 min before being cooled to 30 °C. Na_2_S (1%) in 1% NaHCO_3_ was employed as a reducer prior to the seeding of bacteria in the media (E^0^ of the medium was – 200 mV). The potential was measured with a pH meter: Greisinger G1500. A sterile 10 N solution of NaOH was used to bring the medium's final pH down to 7.5. The proportion of the inoculate volume to the medium volume was 5%. The bacteria were cultivated anaerobically in flasks for 72 h at 37 °C^41^. In addition, reduced FeS and Na_2_S were included in the medium which provided the necessary redox conditions for SRB cultures. Biomass of bacterial cultures was determined photometrically, according to the method previously described in the published paper^39^: about 1 mL of liquid medium without Mohr’s salt was transferred into a plastic cuvette and taken to a biophotometer (Eppendorf BioPhotometerD30) for taring. Subsequently, 1 mL of bacterial suspension was transferred into another cuvette and taken again to the biophotometer. Before SRB were used for the experiments, optical density (OD_340_) was always measured. The optical density of the inoculate was measured and normalized to be the same amount of cells inoculating all bacterial growth.

After 18 h of exponential growth and 36 h of stationary growth, cells were harvested, centrifuged for 30 min at 5000×*g* at 4 °C, washed with 0.02 M Tris–HCl buffer, and then suspended in the same buffer at a ratio of 2–2.5 g wet weight to 10 mL of the same solution. Bacterial cells were broken and homogenized using the ultrasonic disintegrator at 22 kHz for 1 min at 0 °C with subsequent centrifugation (17,000×*g*, 60 min, 4 °C) to remove the cell debris and to obtain cell-free extracts.

### Assays for enzyme activity and kinetic studies

The rate of NADH or NADPH peroxidation was measured at 340 nm (ɛ = 6220 M^−1^ cm^−1^). The addition of 2 mM H_2_O_2_ started the process. The assay mixture contained cell-free extracts extracts diluted in Tris–HCl buffer (50 mM, pH 7.6) (final volume, 1 mL). As previously mentioned^38^, NADH and NADPH were added in quantities ranging from 50 to 500 μM. The control samples were the same mixture, but without bacterial cell-free extracts. The Bradford method was used to calculate the protein content in the cell-free extracts^42^.

A product proportional to the amount of peroxidase activity is produced when H_2_O_2_ and the probe react, and this reaction can be seen using colorimetric (570 nm) and fluorometric (λ_ex_ = 535/λ_em_ = 587 nm) techniques (Sigma-Aldrich kit for the determination of peroxidase activity was used). The amount of peroxidase that lowers 1.0 mol of H_2_O_2_ per min at 37 °C is known as one unit (U = 1 μmol/min). As U × mg^−1^ protein, specific enzyme activity was expressed.

Specific enzyme's activity in cell-free extracts of both bacterial strains were also determined under the influence of two important factor pH values (4.0–10.0) and temperature ranges (20, 25, 30, 35, 40, and 45 °C). Under the various settings (pH and temperature), the composition of the same combination remained essentially the same. pH was adjusted with acid (HCl) and base (NaOH) addition. The temperature of the mixture was also adjusted and cultivated under different temperature regimes.

In a standard incubation medium (discussed above) with adjusted physical and chemical properties of the relevant test parameters (*i.e.*, the incubation duration, substrate concentration, temperature, and pH), kinetic study of the enzyme reaction was carried out. The initial (instantaneous) reaction velocity (*V*_0_), maximum reaction velocity (*V*_max_), the maximum amount of the reaction product (*P*_max_), and the characteristic reaction time (time of half saturation, τ) were identified as the kinetic parameters describing the H_2_O_2_ reaction.

Stoichiometric calculations were used to determine the reaction product's volume. A Lineweaver–Burk plot^43^ was used to establish the kinetic parameters that define peroxidase processes, including the Michaelis constant (*K*_m_) and maximal reaction velocity of the substrate degradation. Initial velocities with various substrate concentrations were obtained under normal assay conditions in order to analyze the substrate kinetic mechanism of peroxidases.

To model the kinetic data for quick equilibrium rate equations, the obtained data were also examined by global curve fitting in SigmaPlot (Systat Software, Inc., https://systatsoftware.com/products/sigmaplot/sigmaplot-how-to-cite-sigmaplot/, Palo Alto, CA 94303, the USA) describing ordered sequential *V* = (*V*_max_ [A] [B])/(K_A_ K_B_ + K_B_ [A] + [A] [B]), and random sequential *V* = (*V*_max_ [A] [B])/(α K_A_ K_B_ + K_B_ [A] + K_A_ [B] + [A] [B]) kinetic mechanisms, where *V* is the initial velocity, V_max_ is the maximum velocity, K_A_ and K_B_ are the *K*_m_ values for substrates A and B, respectively, and α is the interaction factor if the binding of one substrate changes the dissociation constant for the other^44^.

### Statistical analysis

The MS Office and Origin (www.originlab.com) softwares were used to calculate the data' kinetic and statistical properties. The Student t-test was used to analyze the research findings utilizing methods of variation statistics. The method of least squares was used to determine the linear equation. The absolute value of the correlation coefficient r was ranging from 0.90 to 0.98. The significance of the calculated parameters of the line was tested by the Fisher’s F-test. The approximation was deemed accurate approximation when P ≤ 0.05^45^. Overall differences between enzyme activities and kinetic parameters of NADH and NADPH peroxidases were determined by principal component analysis (PCA). Eigenvalue was estimated for two groups to be above zero, explaining 99% of total variance.

### Ethics approval and consent to participate

Authors are confirming that all methods were carried out in accordance with relevant guidelines and regulations. Authors are confirming that all experimental protocols were approved by Masaryk University committee (No. EKV-2021-060). Authors are confirming that informed consent was obtained from all subjects and/or their legal guardian(s).

## Results

**Figure 1:**
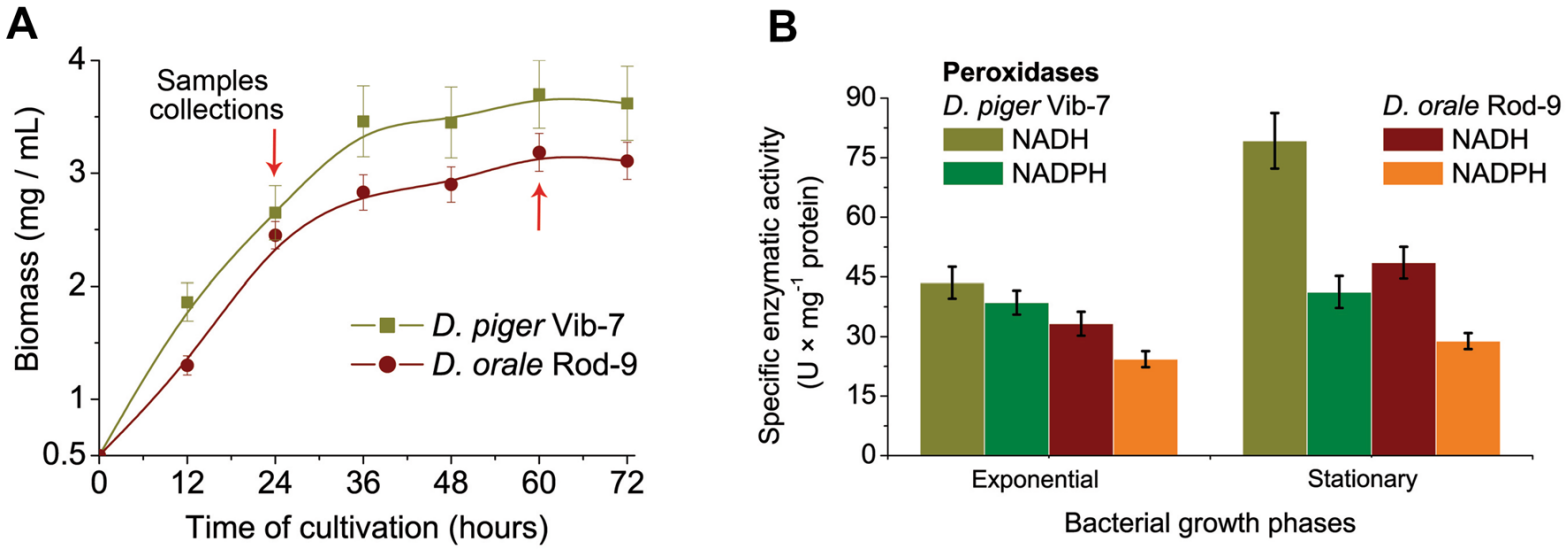
Biomass accumulation of *D. piger* Vib-7 and *D. orale* Rod-9 (**A**) as well as NADH/NADPH peroxidase activity (**B**) in cell-free extract of intestinal SRB depending on their physiological phase (mean ± SD, *n* = 5).

The intestinal SRB *D. piger* Vib-7 reached the growth phase after 36 h in the stationary phase, but *D. orale* Rod-9 had the maximum after 60 h. The sample collection was done from both cultures after 24 h (the exponential phase) and 60 h (the stationary phase) of the cultivation (Fig. 1A). Specific peroxidase activity—which is an important enzyme in the process of antioxidant defense in SRB—was measured in the cell free extract of *D. piger* Vib-7 and *D. orale* Rod-9 cells, taken during the exponential and stationary phase (Fig. 1B). The highest activity of NADH peroxidase (79.25 and 48.55 U × mg^−1^ of protein) was detected among extracts of *D. piger* Vib-7 and *D. orale* Rod-9, cultivated during the stationary phases. Although, the activity of *D. orale* Rod-9 were slightly different between the exponential and stationary phases, the activity of NADPH peroxidases in *D. piger* Vib-7 was similar (p > 0.05) in the exponential and stationary phases.

**Figure 2:**
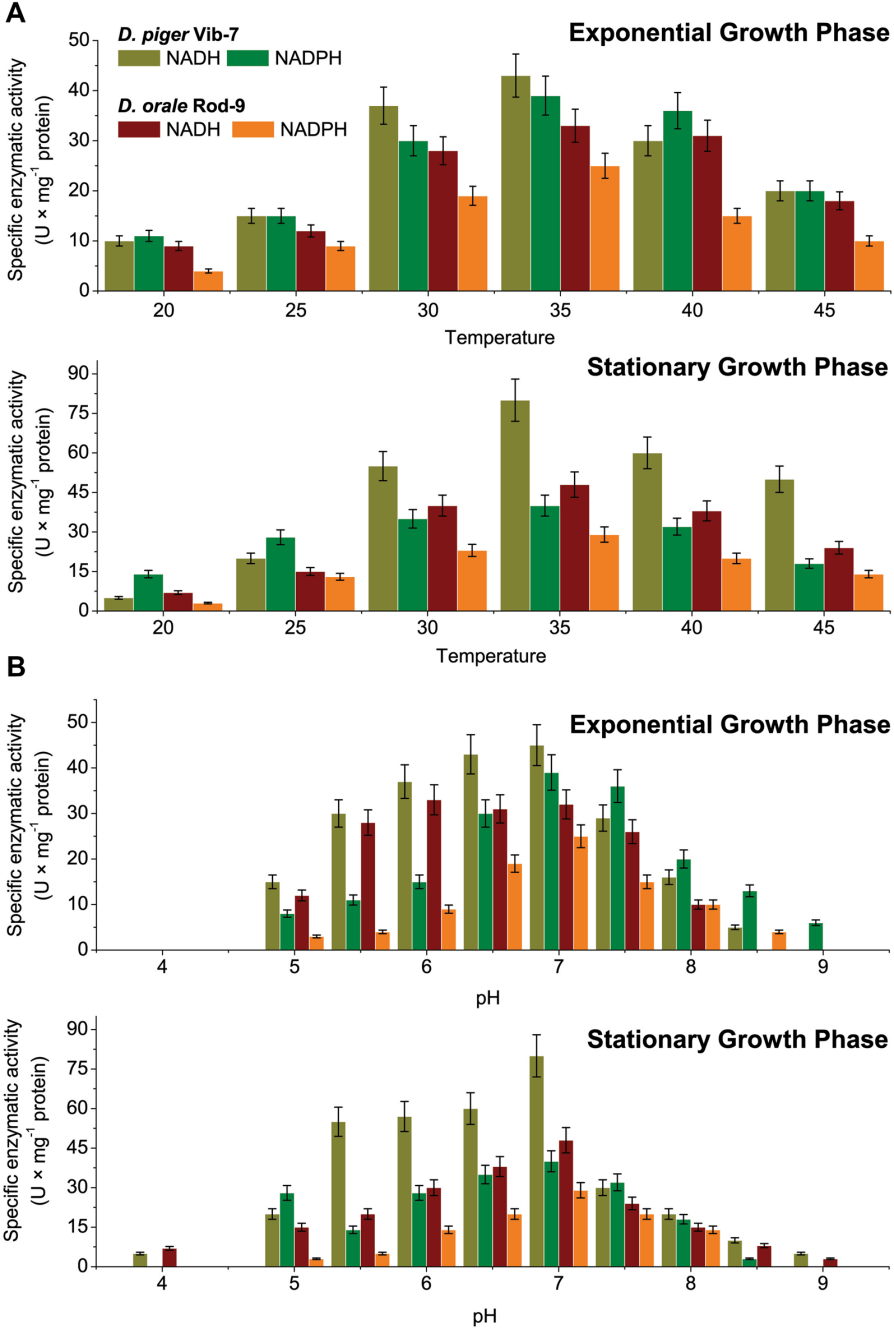
The effect of temperature (**A**) and pH (**B**) on NADH and NADPH peroxidase activity (mean ± SD, *n* = 5) in the cell-free extracts prepared from various bacterial growth phases (exponential and stationary).

Enzymatic activity is heavily influenced by environmental and physical factors, including temperature and pH. In some cases, temperature and pH may simulate the activity and at the same time sometimes dissimulate the activity too. The activities of NADH and NADPH peroxidases in intestinal SRB genus *Desulfovibrio* and *Desulfomicrobium* have never been monitored with scientific accuracy; the effects of temperature and pH of the reaction mixture on peroxidase activity in the cell-free extracts of the SRB was studied (Fig. 2).

The optimal temperature for enzymatic reactions was determined in both bacterial species of *Desulfovibrio* and *Desulfomicrobium* genera and for both enzymes (NADH and NADPH peroxidases) at 35 °C. The increase (max 45 °C) or decrease (min 20 °C) of the optimal temperature resulted in extreme decrease of enzymatic activity in extracts collected during exponential and stationary growth phases. Though, both enzymes showed lower activity at lower temperatures (20–25 °C), during the stationary phase of both tested bacterial cultures, in comparison with results obtained at the optimal temperature (Fig. 2A). The highest enzymatic activity of NADH and NADPH peroxidases for *D. piger* Vib-7 and *D. orale* Rod-9 was measured at pH 6.5 to 7.0. for both enzymes during the stationary and exponential phases. The increase of pH resulted in another extreme reduction of enzymatic activity, where the lowest enzymatic activity was at pH 8, while enzymatic activity of *D. orale* was under the detection limit in the exponential growth phase. A similar trend was observable under the pH conditions lower than 5, where the enzymatic activity was not detectable during the exponential phase in both bacterial strains (Fig. 2B). Obviously, enzymatic activity was inhibited by pH under 5, but in the stationary phase, NADH peroxidase activity was still detectable in very low concentrations (in both bacterial extracts).

Thus, as a function of temperature and pH, enzyme activity often displayed bell-shaped curves. These enzymes functioned the best at 35 °C and a pH of 6.5–7.0, respectively. The enzymatic activity in the cell-free bacterial extracts of the SRB was reduced in response to changes in temperature and pH.

**Figure 3:**
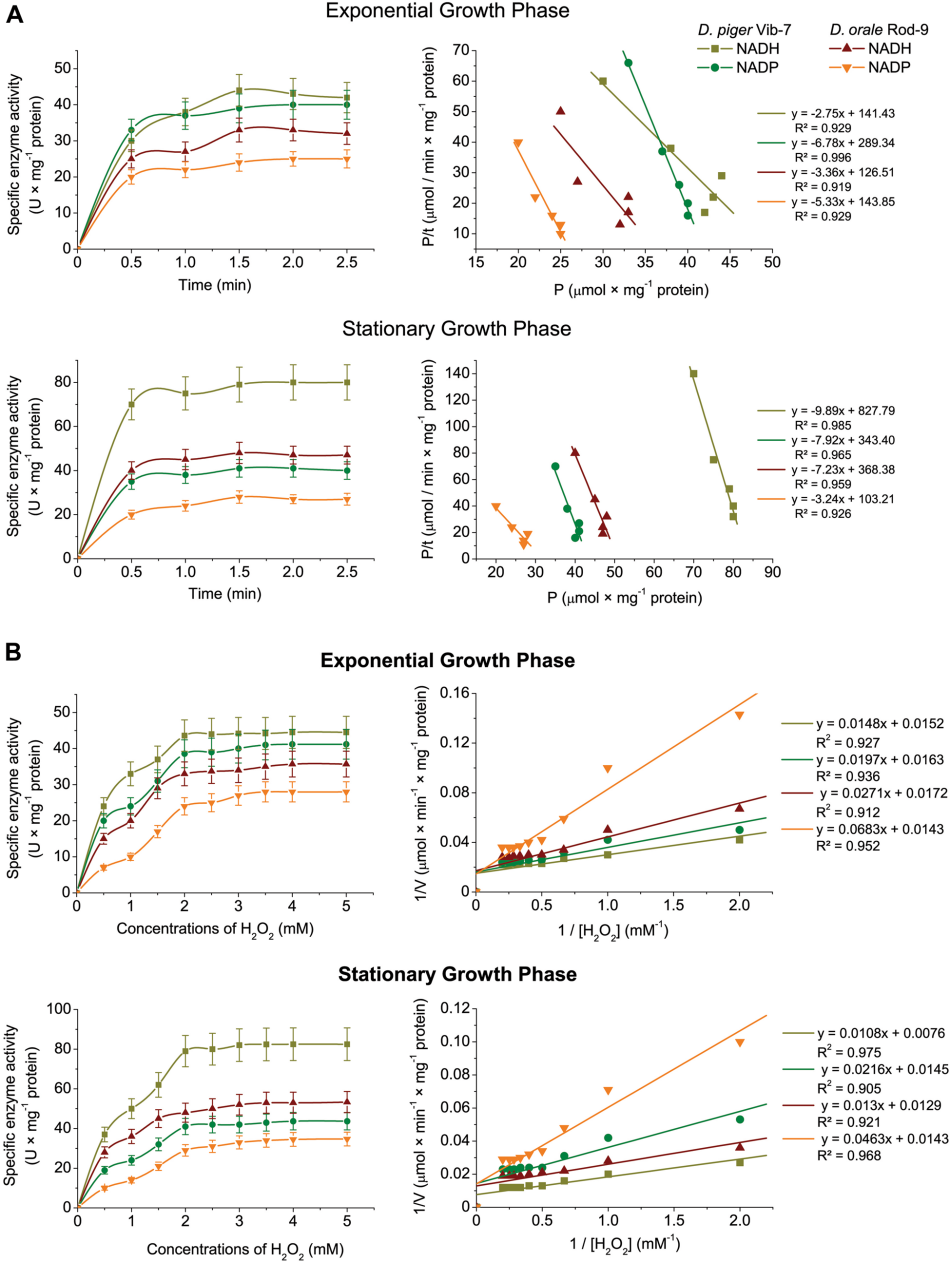
Kinetic parameters of NADH and NADPH peroxidase activity in the cell-free extracts of *D. piger* Vib-7 and *D. orale* Rod-9 prepared from various bacterial growth phases (exponential and stationary): dynamics of product accumulation and its linearization of curves in {P/t; P} coordinates (mean ± SD, *n* = 5; R^2^ > 0.93; F < 0.02) (**A**); the effect of different substrate concentrations (H_2_O_2_) on the enzyme activity and linearization of concentration curves in the Lineweaver–Burk plot, where *V* is velocity of the enzyme reaction and [H_2_O_2_] is substrate concentration (*n* = 5; R^2^ > 0.92; F < 0.005) (**B**).

Investigation of the kinetic characteristics of both types of peroxidases involved the studying of the reaction dynamics product accumulation (Fig. 3). According to experimental evidence, peroxidase activity kinetic curves tend to saturate (Fig. 3A). According to the analysis of the data, the kinetics of peroxidase activity in the sulfate-reducing bacteria was consistent with a zero-order reaction between 0 and 1 min (the graph showing the relationship between product formation and incubation time was nearly linear throughout this time interval).

Therefore, the incubation time for bacterial cell extracts was 2.5 min. In the entire time range during the exponential development phase, *D. piger* Vib-7 cell-free extracts produced more NADH and NADPH peroxidase products than *D. orale* Rod-9 (38 ± 3.52 and 27 ± 2.33 µmol × mg^−1^ protein) (Table 1). When compared to the NADPH peroxidases of the two extracts, NADH peroxidase activity was higher during the first 1.5 min in the stationary phase of both *D. piger* Vib-7 and *D. orale* Rod-9 (Fig. 3A).

**Table 1:** Kinetic parameters of the NADH and NADPH peroxidases from intestinal sulfate-reducing bacteria*

Kinetic parameters	*D. piger* Vib-7		*D. orale* Rod-9	
	NADH	NADPH	NADH	NADPH
Exponential growth phase				
* V*_0_ (µmol × min^-1^ × mg^−1^ protein)	141 ± 13	289 ± 27	127 ± 11	144 ± 13
* P*_max_ (µmol × mg^−1^ protein)	51 ± 4	43 ± 3	38 ± 3	27 ± 2
τ (min)	0.36 ± 0.03	0.15 ± 0.012	0.30 ± 0.017	0.19 ± 0.017
* V*_max_ (µmol × min^−1^ × mg^−1^ protein)	66 ± 5	61 ± 5	58 ± 6	70 ± 6
* K*_m_ (mM)	0.97 ± 0.08	1.21 ± 0.11	1.58 ± 0.13	4.76 ± 0.44
Stationary growth phase				
* V*_0_ (µmol × min^−1^ × mg^−1^ protein)	828 ± 7	343 ± 24	368 ± 34	103 ± 9
* P*_max_ (µmol × mg^−1^ protein)	84 ± 7	43 ± 4	51 ± 4	32 ± 2
τ (min)	0.10 ± 0.09	0.13 ± 0.01	0.14 ± 0.01	0.31 ± 0.02
* V*_max_ (µmol × min^−1^ × mg^−1^ protein)	132 ± 12	69 ± 5	78 ± 6	70 ± 6
* K*_m_ (mM)	1.42 ± 0.11	1.49 ± 0.13	1.02 ± 0.10	3.24 ± 0.31

*V_0_ is initial (instantaneous) reaction rate; *P*_max_ is maximum amount (plateau) of the reaction product; τ is the reaction time (half saturation period), *V*_max_ is maximum rate of the enzyme reaction; K_m_ is Michaelis constant which was determined by substrate (hydrogen peroxide). Statistical significance of the values mean ± SD, *n* = 5; ***P < 0.001, compared to the *D. piger* Vib-7 strain.

By linearizing the data in the P/t; P coordinates, the fundamental kinetic characteristics of the reaction in the sulfate-reducing bacteria were calculated. The concentration of substrate affects how the NADH and NADPH peroxidase activities behave in a kinetic manner (H_2_O_2_). The examined enzyme's activity rose monotonically as H_2_O_2_ concentrations increased from 0.5 to 5 mM, and it remained at the same level (plateau) at substrate concentrations greater than 2.0 mM (Fig. 3B).

The enzyme was saturated with the substrate, and greater doses of H_2_O_2_ (2.0–5.0 mM) had no effect on its activity; it remained constant (plateau). The tangent slope and position at where the dependence 1/V; 1/[S] curves intersect the vertical axis were used to identify them. By linearizing the data in the Lineweaver–Burk plot, the fundamental kinetic parameters of NADH and NADPH peroxidase activity in *D. piger* Vib-7 and *D. orale* Rod-9 were determined. Both strains had considerably different NADH and NADPH peroxidase kinetic parameters: *D. orale* Rod-9 and *D. piger* Vib-7. The maximum amount of the production reaction (P_max_) was used to calculate the enzymes' initial (instantaneous) reaction velocity (V_0_).

As shown in Table 1, *V*_0_ for for NADPH peroxidase reaction was higher in both strains during exponential growth phase in comparison with NADH: *D. piger* Vib-7 (289 ± 27.43 µmol × min^−1^ × mg^−1^ protein) and *D. orale* Rod-9 (144 ± 13.55 µmol × min^−1^ × mg^−1^ protein). Oppositely, the parameter *V*_0_ for NADH peroxidase reaction was more than twice and triplicate higher than NADPH during stationary phase for the both included strains, *D. piger* Vib-7 and *D. orale* Rod-9, respectively. The same observation was seen with product reaction (*P*_max_), both during exponential and stationary growth; *D. piger* Vib-7 had higher NADH and NADPH peroxidase reaction in comparison with *D. orale* Rod-9. The values of the reaction time (τ) during the exponential growth phase was higher (NADH peroxidase reaction, 0.36 ± 0.29 min) for *D. piger* Vib-7, but NADPH was higher for *D. orale* Rod-9 (0.19 ± 0.017 min). *D. orale* Rod-9 had higher the values of the reaction time (τ) for both peroxidase reactions (NADH and NADPH) during the stationary phase in comparison with another tested strain (*D. piger* Vib-7). *V*_max_ values presented with the same tendency, as NADH values were higher in *D. piger* Vib-7 strain, but NADPH for *D. orale* Rod-9 strain; ahough, the differences during the stationary phase are almost neglectable.

Michaelis constant (*K*_m_) was lower during the exponential phase for *D. piger* Vib-7 strain, the both NADH and NADPH. *K*_m_ was higher (NADH peroxidase reaction, 1.42 ± 0.11 mM) during the stationary phase for *D. piger* Vib-7. Low *K*_m_ values indicate a high affinity of the enzyme for the substrate in question. It can be taken into consideration that monitoring *K*_m_ is necessary to differentiate the inhibitory mechanism (competitive or non-competitive type). The described results of the NADH and NADPH reactions and the kinetic properties of the enzyme in cell-free extracts of *D. piger* Vib-7 and *D. orale* Rod-9 strains are novel and have never been reported in the literature before.

**Figure 4:**
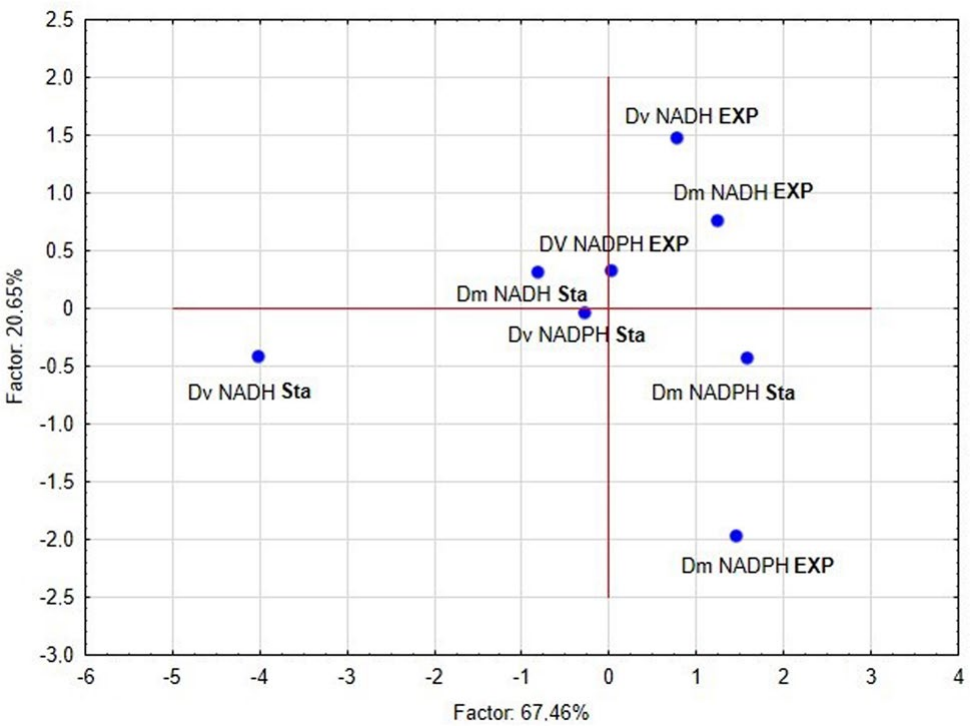
Principal component analysis (PCA) based on kinetic parameters of NADH and NADPH peroxidase activity in the cell-free extracts of *D. piger* Vib-7 (Dv) and *D. orale* Rod-9 (Dm) prepared from various bacterial growth phases (exponential (EXP) and stationary (Sta)).

Principal components analysis (PCA) of all calculated parameters has lead to 5 groups according to the Eigenvalue. The first group: Dm NADH Sta, DvNADPH EXP, Dv NADPH Sta and Dm NADPH Sta; the second group: Dv NADH EXP and Dm NADH EXP; the third group: Dv NADH Sta; the fourth group: Dm NADPH Sta; the fifth group: Dm NADPH EXP. Exponential growth phases are mainly separated from the stationary growth phases (only DvNADPH EXP was in the group with the stationary growth phases) (Fig. 4).

## Discussion

Antioxidant enzymes are endogenous systems can scavenge ROS^30^. SOD, catalase, and peroxidases belong to antioxidant enzymes that are able to prevent intracellular ROS formation, same as lipid peroxidation^46^. While catalase breaks down superoxide radicals into water and O_2_, superoxide dismutase is able to convert superoxide radicals into H_2_O_2_^28^.

Heme-containing enzymes called peroxidases (EC 1.11.1.7; hydrogen peroxide oxidoreductase) can catalyze the oxidation of substrates utilizing H_2_O_2_^47^, a non-radical ROS product of regular aerobic metabolism. The high amount of H_2_O_2_ could be converted into more reactive ROS (e.g., hydroxyl radicals capable of oxidizing biomolecules) that can damage tissue, cause cell death or carcinogenesis, aging and have an effect on cardiovascular diseases. Therefore, a crucial component of overall antioxidant activity is H_2_O_2_ scavenging activity^48, 49^.

The discovery of new species of SRB resulted in significant changes in their taxonomy, the same as in concepts about SRB metabolism. Nowadays, many SRB are considered to be aerotolerant anaerobes, and O_2_ might play an important physiological role in the metabolism of SRB. It is suggested that SRB use O_2_ as a final acceptor of electrons during the synthesis of ATP^50, 51^. It should be also noted that O_2_ inhibits some of the critical enzymes of SRB, like lactate dehydrogenase and NAD dependent alcohol dehydrogenase^52^.

Some SRB are partially aerotolerant to some degree^53–56^, and even after prolonged exposure to O_2_, some SRB species can resume anoxic growth. These aerotolerant SRB mainly contain SOD and catalase^55, 57, 58^. There is not much information gained by previous experiments about enzymes involved in O_2_ consumption by SRB. The O_2_ reduction chain consisting of an NADH oxidase (NADH rubredoxin oxidoreductase), which is present in *D. gigas*^59, 60^. NADH oxidase activities have also been found in *D. desulfuricans* NCIB 8301^57^ and *D. vulgaris* Hildenborough^60^. Hardy and Hamilton^55^ found O_2_ reduction activities in several *D. vulgaris*, though NADH oxidase activity was not detected. According to the previous work, it was observed that O_2_ reduction in SRB species takes places in the periplasm and is linked to cytochrome *c*_3_, as Postgate^61^ proposed for *D. desulfuricans* strain. Probably at least three independent systems are able to reduce O_2_ in *D. desulfuricans* CSN. Out of those three systems, one system is active only at low O_2_ concentrations, but it is inhibited by high O_2_ concentrations; the rest two systems are active at all O_2_ concentrations^38^.

The suggestion that the former system is probably active in the periplasm, and it is linked to cytochrome *c*_3_. Oxidation activities of NADH and NADPH are in soluble cell extracts at all O_2_ concentrations and these activities declined with a decrease of O_2_ concentration. NADH oxidase activity was present in all strains that were examined, they all responded similarly to changes in O_2_ concentrations^32, 61^.

These results suggest that the SRB contain NADH oxidase that able to reduce O_2_ directly to H_2_O, though some SRB strains can reduce O_2_ to H_2_O_2_; H_2_O_2_ is further reduced to H_2_O, as these strains possess the NADH peroxidase. Examined *D. desulfuricans* strains contained NADH and NADPH oxidases and peroxidases^38^, therefore these findings correspond with our research that included *D. piger* Vib-7 and *D. orale* Rod-9 where the activity of these enzymes were also observed.

In the research by van Niel et al., these strains do not accumulate polyglucose during growth with various substrates. These properties distinguish them from the following strains: *D. salexigens* and *D. gigas.* In all cases, the addition of substrates resulted in a rapid decline of NADH oxidase activity. The same observation was seen with NADPH oxidase, NADH peroxidase, and NADPH peroxidase, same as with O_2_ consumption by the whole cells of *D. desulfuricans* strains. It is possible that reactive intermediates—which damage the enzyme—are formed during oxidation reactions with O_2_. The more extensive will be the damage and thus inactivation when the faster oxidation is taking place^38^.

The facultative anaerobic bacterium *Lactobacillus casei* IGM394 strains carry several genes that are allowing the strain to tolerate O_2_ and reactive oxygen species (ROS), though the complete functions have not been revealed yet. The decreased growth and high H_2_O_2_ accumulation were observed in the NADH peroxidase in comparison with wild types. Due to the H_2_O_2_ degradation capacity, it was revealed that NADH peroxidase is a major H_2_O_2_ degrading enzyme in *L. casei* IGM394. Conversely, the H_2_O_2_ tolerance mechanism is dependent only on NADH peroxidase in *L. casei* IGM394^62^.

The inactivation in this organism is considered to be an intrinsic property of the enzymes and their substrates. Inactivation only happens in the case when both oxidizable substrates and O_2_ are present. According to the results, the enzyme–substrate complex is prone to denaturation. *Enterococcus faecalis* NADH oxidases are one of the best studied. *D. gigas*^60^ and *E. faecalis*^63^ NADH oxidases both contain FAD, no cental metal ions and they are sensitive to sulfhydryl agents. The gradual removal of FAD is the reason for the instability of *S. faecalis* NADH oxidase^64^. Although the addition of FAD to the cell extracts increased the rate of oxidation of NADH, but it had no effect on the inactivation rate. The experiments were conducted also on the mutant strain where NADH peroxidase activity was observed, but not in the wild type. NADH peroxidase activities in in vitro systems containing NADH hydrogen peroxide, and a bacterial NADH oxidoreductase from *Desulfovibrio vulgaris* and from *Clostridium perfringens* was also shown^65^.

*Desulfovibrio* species (SRB group) are ubiquitous anaerobic microorganisms and they have a large metabolic diversity^4, 18, 22, 66–68^. All SRB members are unified because they use sulfate as the terminal electron acceptor, reducing it to H_2_S. Though, SRB species are classified as strict anaerobes, they are able to deal with the temporary presence of O_2_ (in natural habitats, such as marine surface waters, microbial mats, sewers, rice paddies and oil pipelines), and several *Desulfovibrio* species was found to be able to oxidize organic substrates under low levels of O_2_^69^. Otherwise, *Desulfovibrio* that are aerotolerant cannot utilize O_2_ for growth^50^, therefore, in the presence of sulfide, SRB are more O_2_ sensitive^53^.

To obtain more insights with regard to peroxidases in SRB, we examined the genomes of closely-related organisms. However, despite our efforts to ascertain more concrete information, the findings are the following. Only one refseq genome for *Desulfomicrobium orale* (https://www.ncbi.nlm.nih.gov/nuccore/NZ_CP014230.1) is available at NCBI. *Desulfomicrobium orale* genes were found to encode for a putative Dyp-type peroxidase (WP_066605144.1), a putative thiol-peroxidase (WP_066607718.1), a putative superoxide dismutase (WP_066603707.1), and two putative peroxireducins (WP_066605219.1; WP_066606413.1). However, which is more relevant to our study, several oxidoreductases are encoded in the genome, such as two putative FAD-dependent oxidoreductases (WP_066608725.1; WP_066606935.1; WP_066605517.1), two NAD(P)/FAD-dependent oxidoreductases (WP_066604797.1; WP_151192312.1), and two NAD(P)-dependent oxidoreductases (WP_066606986.1; WP_066607102.1). From our perspective, it might be reasonable to consider that the last five enzymes could play a role in the specific enzyme activities under investigation. Nevertheless, the enzyme traits predicted by the automated genome annotation pipeline might not necessarily suggest a direct involvement of these enzymes in peroxidase functions. Identifying the enzyme characteristics would entail the cloning, expression, and characterization of each of the presumed enzymes. Alternatively, another approach could involve pinpointing the pertinent putative peroxidases through transcriptomics within specific growth experiments. Both routes would be interesting to follow in future studies. Still, our study does not primarily concern the genetic properties of the examined enzymes through a bioinformatics approach. There is also a genome of a closely related *Desulfovibrio piger* available at NCBI. This genome is not a refseq genome. While we made an effort to locate the information, we did not explore the automated annotation pipeline results for this genome. Moreover, we conducted an additional literature search to determine whether peroxidase activities had previously been reported for other SRB. Regrettably, no records pertaining to this aspect were found. Hence, the activity of NADH and NADPH peroxidases has not been tested till now, too. There is an overall scarcity about this information on peroxidases in environmental SRB. Regarding environmental SRB, the presence of peroxidases might be contingent upon the specific strain or species under investigation. Some SRB may possess peroxidases as part of their metabolic pathways, while others may rely on alternative enzymes or mechanisms for managing oxidative stress.

## Conclusions

NADH and NADPH peroxidases are important enzymes that are putatively involved in the antioxidant defense systems of intestinal SRB. Both peroxidases could represent an evolutionary response to oxidative stress, and they can sustain their activity over a broad range of temperature and pH conditions, support the process of dissimilatory sulfate reduction, and the production of H_2_S. At 35 °C and pH 7.0, the maximal specific enzymatic activities were found.

In the case of *D. piger* Vib-7 compared to *D. orale* Rod-9, the kinetic parameters of enzyme specific activity, including *V*_0_ and *V*_max_, were significantly higher. The concentrations of the substrate (H_2_O_2_) affected the kinetic parameters of enzyme reactions. Between *D. piger* Vib-7 and *D. orale* Rod-9, the *K*_m_ values were notably different throughout the exponential and stationary growth phases. The NADH peroxidase of *D. piger* Vib-7 is the exception to this finding. *D. orale* Rod-9 displayed much higher *K*_m_ during both phases.

As *D. piger* Vib-7 and *D. orale* Rod-9 peroxidases could be involved in their respective metabolisms as well as in the dissimilatory sulfate reduction and the production of H_2_S, the results obtained may be considered as significant insights and perspectives for clarification of the etiological role in the development of bowel diseases in humans and animals.

## Data Availability

The datasets used and/or analyzed during the current study are available from the corresponding author on reasonable request.

## References

[1] Rowan FE, Docherty NG, Coffey JC, O’Connell PR (2009). Sulphate-reducing bacteria and hydrogen sulphide in the aetiology of ulcerative colitis. Br. J. Surg..

[2] Pitcher MC, Cummings JH (1996). Hydrogen sulphide: A bacterial toxin in ulcerative colitis?. Gut.

[3] Florin THJ, Gibson GR, Neale G, Cummings JH (1990). A role for sulfate reducing bacteria in ulcerative colitis. Gastroenterology.

[4] Kováč J, Vítězová M, Kushkevych I (2018). Metabolic activity of sulfate-reducing bacteria from rodents with colitis. Open Med..

[5] Kushkevych I (2019). Hydrogen sulfide effects on the survival of lactobacilli with emphasis on the development of inflammatory bowel diseases. Biomolecules.

[6] Kushkevych I, Dordević D, Kollar P, Vítězová M, Drago L (2019). Hydrogen sulfide as a toxic product in the small-large intestine axis and its role in IBD development. JCM.

[7] Kushkevych I (2020). Evaluation of physiological parameters of intestinal sulfate-reducing bacteria isolated from patients suffering from IBD and healthy people. JCM.

[8] Dordević D, Jančíková S, Vítězová M, Kushkevych I (2020). Hydrogen sulfide toxicity in the gut environment: Meta-analysis of sulfate-reducing and lactic acid bacteria in inflammatory processes. J. Adv. Res..

[9] Paulo LM, Stams AJM, Sousa DZ (2015). Methanogens, sulphate and heavy metals: A complex system. Rev. Environ. Sci. Biotechnol..

[10] Singh S, Lin H (2015). Hydrogen sulfide in physiology and diseases of the digestive tract. Microorganisms.

[11] Cummings JH, Macfarlane GT, Macfarlane S (2003). Intestinal bacteria and ulcerative colitis. Curr. Issues Intest. Microbiol..

[12] Gibson GR, Macfarlane GT, Cummings JH (1988). Occurrence of sulphate-reducing bacteria in human faeces and the relationship of dissimilatory sulphate reduction to methanogenesis in the large gut. J. Appl. Bacteriol..

[13] Kushkevych I, Coufalová M, Vítězová M, Rittmann SK-MR (2020). Sulfate-reducing bacteria of the oral cavity and their relation with periodontitis—recent advances. JCM.

[14] Kushkevych I, Dordević D, Vítězová M (2021). Possible synergy effect of hydrogen sulfide and acetate produced by sulfate-reducing bacteria on inflammatory bowel disease development. J. Adv. Res..

[15] Kushkevych I (2020). Recent advances in metabolic pathways of sulfate reduction in intestinal bacteria. Cells.

[16] Kushkevych I (2017). Kinetic properties of growth of intestinal sulphate-reducing bacteria isolated from healthy mice and mice with ulcerative colitis. Acta Vet. Brno.

[17] Abdulina D, Kováč J, Iutynska G, Kushkevych I (2020). ATP sulfurylase activity of sulfate-reducing bacteria from various ecotopes. 3 Biotech.

[18] Kuever J, Rosenberg E, DeLong EF, Lory S, Stackebrandt E, Thompson F (2014). The family Desulfovibrionaceae. The Prokaryotes.

[19] Kushkevych I, Kováč J, Vítězová M, Vítěz T, Bartoš M (2018). The diversity of sulfate-reducing bacteria in the seven bioreactors. Arch. Microbiol..

[20] Kushkevych I, Blumenberg M, Shaaban M, Elgaml A (2020). Isolation and purification of sulfate-reducing bacteria. Microorganisms.

[21] Kushkevych I (2019). The sulfate-reducing microbial communities and meta-analysis of their occurrence during diseases of small-large intestine axis. JCM.

[22] Brenner, D. J., Krieg, N. R., Staley, J. T. & Garrity, G. M. The proteobacteria, part C: The alpha-, beta-, delta-, and epsilonproteobacteria. in *Bergey’s Manual of Systematic Bacteriology* (Springer, 2005).

[23] Postgate JR (1959). Sulphate reduction by bacteria. Annu. Rev. Microbiol..

[24] Kushkevych IV (2015). Kinetic properties of pyruvate ferredoxin oxidoreductase of intestinal sulfate-reducing bacteria Desulfovibrio piger Vib-7 and Desulfomicrobium sp. rod-9. Pol. J. Microbiol..

[25] Barton, L. L. & Fauque, G. D. Biochemistry, physiology and biotechnology of sulfate‐reducing bacteria. in *Advances in Applied Microbiology*, vol. 68, 41–98 (Elsevier, 2009). 10.1016/S0065-2164(09)01202-7 19426853

[26] Fauque, G. D. Ecology of sulfate-reducing bacteria. in *Sulfate-Reducing Bacteria* (ed. Barton, L. L.) 217–241 (Springer, 1995). 10.1007/978-1-4899-1582-5_8.

[27] Gülçin İ, Bursal E, Şehitoğlu MH, Bilsel M, Gören AC (2010). Polyphenol contents and antioxidant activity of lyophilized aqueous extract of propolis from Erzurum, Turkey. Food Chem. Toxicol..

[28] TejeraGarcía NA, Iribarne C, Palma F, Lluch C (2007). Inhibition of the catalase activity from *Phaseolus vulgaris* and *Medicago sativa* by sodium chloride. Plant Physiol. Biochem..

[29] Nóbrega CS, Pauleta SR (2019). Reduction of hydrogen peroxide in gram-negative bacteria–bacterial peroxidases. Adv. Microb. Physiol..

[30] Bursal E (2013). Kinetic properties of peroxidase enzyme from chard ( *Beta** vulgaris* Subspecies *cicla* ) leaves. Int. J. Food Prop..

[31] Pyo Y-H, Lee T-C, Logendra L, Rosen RT (2004). Antioxidant activity and phenolic compounds of Swiss chard (*Beta vulgaris* subspecies cycla) extracts. Food Chem..

[32] La Carbona S (2007). Comparative study of the physiological roles of three peroxidases (NADH peroxidase, alkyl hydroperoxide reductase and Thiol peroxidase) in oxidative stress response, survival inside macrophages and virulence of *Enterococcus faecalis*. Mol. Microbiol..

[33] Miller H, Poole LB, Claiborne A (1990). Heterogeneity among the flavin-containing NADH peroxidases of group D streptococci. Analysis of the enzyme from *Streptococcus faecalis* ATCC 9790. J. Biol. Chem..

[34] Stehle T, Claiborne A, Schulz GE (1993). NADH binding site and catalysis of NADH peroxidase. Eur. J. Biochem..

[35] Yeh JI, Claiborne A (2002). Crystal structures of oxidized and reduced forms of NADH peroxidase. Methods Enzymol..

[36] Gordon J, Holman RA, McLeod JW (1953). Further observations on the production of hydrogen peroxide by anaerobic bacteria. J. Pathol..

[37] Conn EE, Kraemer LM, Liu PN, Vennesland B (1952). The aerobic oxidation of reduced triphosphopyridine nucleotide by a wheat germ enzyme system. J. Biol. Chem..

[38] van Niel EWJ, Gottschal JC (1998). Oxygen consumption by *Desulfovibrio* strains with and without polyglucose. Appl. Environ. Microbiol..

[39] Kushkevych I (2013). Identification of sulfate-reducing bacteria strains of human large intestine. Biol. Stud..

[40] Postgate J (1984). The Suphate-Reducing Bacteria.

[41] Kovac J, Kushkevych I (2019). New modification of cultivation medium for isolation and growth of intestinal sulfate-reducing bacteria. MendelNet.

[42] Bradford MM (1976). A rapid and sensitive method for the quantitation of microgram quantities of protein utilizing the principle of protein-dye binding. Anal. Biochem..

[43] Lineweaver H, Burk D (1934). The determination of enzyme dissociation constants. J. Am. Chem. Soc..

[44] Segel IH (1976). Biochemical Calculations: How to Solve Mathematical Problems in General Biochemistry.

[45] Bailey NTJ (1995). Statistical Methods in Biology.

[46] Gülçin I (2009). Melatonin administration increases antioxidant enzymes activities and reduces lipid peroxidation in the rainbow trout (*Oncorhynchus mykiss*, Walbaum) erythrocytes. Turk. J. Vet. Anim. Sci..

[47] Manu BT, Rao UJSP (2009). Calcium modulated activity enhancement and thermal stability study of a cationic peroxidase purified from wheat bran. Food Chem..

[48] Gulcin I, Tel AZ, Kirecci E (2008). Antioxidant, antimicrobial, antifungal, and antiradical activities of Cyclotrichium Niveum (BOISS) Manden and Scheng. Int. J. Food Prop..

[49] Ma X, Li H, Dong J, Qian W (2011). Determination of hydrogen peroxide scavenging activity of phenolic acids by employing gold nanoshells precursor composites as nanoprobes. Food Chem..

[50] Dilling W, Cypionka H (1990). Aerobic respiration in sulfate-reducing bacteria*. FEMS Microbiol. Lett..

[51] Lemos RS (2001). The ‘strict’ anaerobe *Desulfovibrio gigas* contains a membrane-bound oxygen-reducing respiratory chain. FEBS Lett..

[52] Krekeler D, Teske A, Cypionka H (1998). Strategies of sulfate-reducing bacteria to escape oxygen stress in a cyanobacterial mat. FEMS Microbiol. Ecol..

[53] Cypionka H, Widdel F, Pfennig N (1985). Survival of sulfate-reducing bacteria after oxygen stress, and growth in sulfate-free oxygen-sulfide gradients. FEMS Microbiol. Lett..

[54] Fukui M, Takii S (1990). Survival of sulfate-reducing bacteria in oxic surface sediment of a seawater lake. FEMS Microbiol. Lett..

[55] Hardy JA, Hamilton WA (1981). The oxygen tolerance of sulfate-reducing bacteria isolated from North Sea waters. Curr. Microbiol..

[56] Risatti JB, Capman WC, Stahl DA (1994). Community structure of a microbial mat: The phylogenetic dimension. Proc. Natl. Acad. Sci. USA.

[57] Abdollahi H, Wimpenny JWT (1990). Effects of oxygen on the growth of *Desulfovibrio desulfuricans*. J. Gen. Microbiol..

[58] Traore AS, Hatchikian CE, Belaich JP, Le Gall J (1981). Microcalorimetric studies of the growth of sulfate-reducing bacteria: energetics of *Desulfovibrio** vulgaris* growth. J. Bacteriol..

[59] Chen L (1993). Rubredoxin oxidase, a new flavo-hemo-protein, is the site of oxygen reduction to water by the ‘strict anaerobe’ *Desulfovibrio** gigas*. Biochem. Biophys. Res. Commun..

[60] Chen L (1993). Purification and characterization of an NADH-rubredoxin oxidoreductase involved in the utilization of oxygen by *Desulfovibrio** gigas*. Eur. J. Biochem..

[61] Postgate JR (1956). Cytochrome c3 and desulphoviridin; pigments of the anaerobe Desulphovibrio desulphuricans. J. Gen. Microbiol..

[62] Naraki S, Igimi S, Sasaki Y (2020). NADH peroxidase plays a crucial role in consuming H_2_O_2_ in *Lactobacillus **casei* IGM394. Biosci. Microb. Food Health.

[63] Schmidt H-L, Stocklein W, Danzer J, Kirch P, Limbach B (1986). Isolation and properties of an H2O-forming NADH oxidase from *Streptococcus faecalis*. Eur. J. Biochem..

[64] Hoskins DD, Whiteley HR, Mackler B (1962). The reduced diphosphopyridine nucleotide oxidase of Streptococcus faecalis: Purification and properties. J. Biol. Chem..

[65] Coulter ED, Shenvi NV, Kurtz DM (1999). NADH peroxidase activity of rubrerythrin. Biochem. Biophys. Res. Commun..

[66] Kushkevych I, Dordević D, Vítězová M (2019). Analysis of pH dose-dependent growth of sulfate-reducing bacteria. Open Med..

[67] Kushkevych IV (2015). Activity and kinetic properties of phosphotransacetylase from intestinal sulfate-reducing bacteria. Acta Biochim. Pol..

[68] Kushkevych I, Kos J, Kollar P, Kralova K, Jampilek J (2018). Activity of ring-substituted 8-hydroxyquinoline-2-carboxanilides against intestinal sulfate-reducing bacteria Desulfovibrio piger. Med. Chem. Res..

[69] Dannenberg S, Kroder M, Dilling W, Cypionka H (1992). Oxidation of H2, organic compounds and inorganic sulfur compounds coupled to reduction of O2 or nitrate by sulfate-reducing bacteria. Arch. Microbiol..

